# Online survey among maritime pilots: job-related stress and strain and the effects on their work ability

**DOI:** 10.1186/s12995-021-00322-2

**Published:** 2021-08-25

**Authors:** Marcus Oldenburg, Jan Herzog, Filip Barbarewicz, Volker Harth, Hans-Joachim Jensen

**Affiliations:** 1 Institute for Occupational and Maritime Medicine Hamburg (ZfAM), University Medical Center Hamburg-Eppendorf (UKE), Seewartenstrasse 10, 20459 Hamburg, Germany; 2 University of Applied Sciences, Flensburg, Germany

**Keywords:** Maritime pilot, Seafaring, Work-related stress, Strain, Daily sleepiness, Work ability

## Abstract

**Background:**

Maritime pilots often navigate ships through challenging waterways. The required 24 h standby rotation system (ROS) poses a stressful working situation. This study aims to describe the current job-related stress and strain among maritime pilots and the effects on their work ability, taking into account the different rotation systems.

**Methods:**

Within a cross-sectional survey, pilots of all German pilots’ associations were asked to complete an online questionnaire. The 1-week ROS (port pilots) was compared with the 4-month ROS (sea and canal pilots). The pilots’ subjective perception of stress and strain was assessed using an established ship-specific questionnaire. Daily sleepiness and work ability were examined respectively using the Epworth Sleepiness Scale (ESS) and the Work Ability Index (WAI).

**Results:**

The study group consisted of 401 male German pilots with an average age of 48.5 years (participation rate 46.9%). More than 50% of the pilots evaluated irregular working hours as the main stressor in their job. 79.8% of the pilots (especially 4-month ROS) experienced high psychological demands in their workplace. 83.3% stated having regularly neglected their private obligations due to job assignments. Pilots from the 4-month ROS experienced insufficiently predictable free time and long operation times at a stretch as stressors (*p* < 0.001 and *p* = 0.037). Elevated daily sleepiness was found in 41.9% of the pilots. The overall evaluation of the WAI questionnaire showed good to very good work ability at 77.3%. Additionally, no significant differences in the daily sleepiness or the work ability were observed between the pilots of the different two ROS.

**Conclusions:**

Due to their subjectively higher job-related mental demands, their disturbed work-life balance, and their long operation times at a stretch, it is likely that pilots from the 4-month ROS have significantly higher job stress compared to those in the 1-week ROS. However, this does not lead to more sleepiness or reduced work ability, which suggests that the pilots of this ROS are highly adapted to their working situation. Nevertheless, intervention measures with shortened ROS amongst sea and canal pilots’ associations should be tested in respect of benefit, practicability and acceptance by the pilots.

## Background

Worldwide shipping and the work of maritime pilots go hand in hand as pilots are the ones who navigate ships through challenging or restricted waterways [1]. Their assignments vary in terms of length and working environment and are often characterized by isolation due to limitations of their personal responsiveness within their irregular working shifts [2]. “Fatigue” is also a common problem within the pilot profession, particularly during frequent operations at chronobiologically unfavorable times of day (in prolonged night assignments) or when pilots sometimes have to perform monotonous activities with no special occurrences during the whole pilotage. Already in the 1980s, Cook and Shipley described a significantly increased risk of falling asleep for pilots due to their low activity level [3]. Limited alertness can have far-reaching consequences up to potential life-threatening injuries, death or environmental damage [4]. Main and Chambers [5] stated that so far little attention has been paid to this stressful profession compared to other professions. According to the authors, particularly the (physical) health and, crucially, the (psychological) well-being of this occupational group have been insufficiently investigated.

Pilotage is a freelance activity [6, 7]. Due to the irregular distribution, the working hours of a pilot vary strongly and are not subject to predictable rules. Their duration can greatly exceed common working hours. The German Pilot Act requires a constant availability of pilots 365 days a year at any time of day or night, stringently monitored by regional regulators [8]. Each ship must receive a pilot within specific shipping areas prescribed in the Maritime Pilot Act [6]. To ensure a constant availability of pilots, a fixed system has been established since the seventeenth century. This system works in a similar way to that at a taxi rank: The pilot at the top of the list is allocated to the next ship to be piloted. Upon finishing their service aboard, the pilots return to the end of this list and then move forward step by step until they reach position one of this list again.

The specific rotation system (ROS) used within the German pilots’ associations can differ considerably, depending on the district. The most common systems last for 1 week or 4 months. The port pilots use a 1-week ROS, which provides 8 days on duty (working time) and 6 days off duty alternately, whereby each pilot is assigned to a fixed group. The (retrieval) order within these groups changes according to a fixed system, as does the order of the groups themselves. The ROS of sea and channel pilots’ associations is similar, but based on a 4-month exchange. A 4-month working period (with two to four free days per month) is followed by a 3 to 4-week leave period. Should the number of absent pilots become too large, these pilots’ associations can impose a ban on leave or days off. It can be assumed that these two ROS could cause different levels of work-related stress and strain for the pilots.

The number of ships ordering pilots is subject to seasonal, daily, tidal, weather and cyclical fluctuations [9]. All ships must always be served without delay. The irregular arrival of the ships at the transfer stations makes forward-looking assignment planning and the anticipation of deployment requirements difficult. The length of on-call time between two operations depends mainly on traffic and therefore varies widely. During their standby service, pilots are either at a pilot station or, possibly, at home. How long a pilot can remain in a resting phase is often unpredictable. When a pilot is called up, transport to the ship is usually by pilot boat, taxi or on foot. Overall, the current deployment system requires a high degree of flexibility from pilots and their families.

It is assumed that the high job-related stress and strain pilots experience is likely to impact their health and their work ability. The few available studies about this occupation suggest reduced psychophysical health among pilots, including sleeping disorders [10], cognitive disorders and alertness limitations [2] that may lead to accidents caused by human error [11]. Thus, it is crucial to examine the work ability of pilots. However, no study is available on the current psychophysical stress situation or work capacity of this professional group. Therefore, the purpose of the present survey is to describe the current job-related stress and strain among maritime pilots and the effects on their work ability, taking into account the different rotation systems.

## Methods

### Study sample

In this study, all nine German pilots’ associations were invited to participate. Eight associations and hence 855 employed pilots agreed to take part (from 10 to 65%). One pilots’ association rejected the participation completely. Out of the 855 pilots, 401 participated in this study (46.9%). Participation was voluntary, and the data collected in the course of the research project were subject to secrecy and the provisions of data protection law. All participants gave their informed consent before taking part in this study. There was a positive ethics vote by the Hamburg Medical Association (PV No. 5498).

### Questionnaires

An anonymous query was carried out via an encrypted online portal (LamaPoll) for easy processing. The online questionnaire asked about demographic and lifestyle parameters, main job-related stressors, work-life balance, accidents throughout the pilots’ working life, health aspects and retirement. As the online portal used did not allow questions to be skipped, there are no missing data (5.4% started their questionnaire but did not complete it).

To assess the stress and strain of maritime occupations a pilot-specific questionnaire was developed basing on an established seafaring specific questionnaire [12]. This was about seafarers’ stress and strain and has previously been used several times in various maritime studies [13, 14]. In this study, the pilots’ subjectively most important stressors were queried. Participants were asked to choose from a list of 10 relevant stressors (based on previous interviews with pilots) up to four that applied to them. In addition, the pilots had the opportunity to add another important stressor.

The questionnaire of this study was created by the heads of some pilots’ associations and by several pilots. Finally, it was tested and improved in a pilot study with 15 employees. In the online portal, all pilots had the opportunity to comment and to add some further aspects in free text. Sufficiently high representativeness was assumed for the assessment of the pilots’ stress and strain in view of this supplementary free text plus the fact that several pilots were involved and the aforementioned maritime specific questionnaire was used as a basis. For reliability analysis, Cronbach’s alpha was calculated to assess the internal consistency of this questionnaire. The internal consistency of the questionnaire is satisfying, with a Cronbach’s alpha of 0.76.

Furthermore, the Epworth Sleepiness Scale (ESS) and the Work Ability Index (WAI) were used as standardized questionnaires:

#### Epworth Sleepiness Scale (ESS)

The Epworth Sleepiness Scale is a standardized method of recording daytime sleepiness using a short questionnaire and has already been used in several studies with seagoing personnel such as bridge officers and sailors [15, 16]. This method is primarily used in sleep medicine for the diagnosis of sleep disorders. The retrospective question is the subjective assessment of a person’s probability of actually falling asleep in eight given typical situations. The questions relate to the normal daily life in recent times [17, 18]. The retest reliability in healthy controls after 5 months is 0.82 (mean difference = 0.2 +/− 2.3) [17, 18].

#### Work Ability Index (WAI)

This questionnaire is used to record work ability and work-related health risks. It is a diagnostic tool for assessing individual work ability, also used as an evaluation instrument for the effectiveness of preventive measures. Points are awarded for certain answers, and the questionnaire is evaluated by adding the individual scores [19, 20]. The reliability and internal consistency of this questionnaire is 0.58 respectively 0.78 [21, 22].

### Data analysis

All statistical evaluations were performed using the program SPSS for Windows (version 25, IBM Corporation). In addition to descriptive statistics (mean with standard deviation), statistical tests were applied (test for normal distribution by Shapiro-Wilk test). For parametric data, the Student’s T test was carried out. Furthermore, the Chi-square test was applied to compare frequencies of two samples. In those Chi-square tests with small numbers, the Fisher’s exact test was used instead. The error probability was set to 5%. The crude odds ratio (OR) including 95% confidence intervals was calculated by binary logistic regression. For adjustment reasons, age, working years as a pilot, examination time (during working phase vs. vacation phase) and ESS were added.

## Results

### Demographic data of the studied group

The average age in the total sample was 48.5 years (SD 8.4 years), and the average BMI 27.3 (SD 3.6). Two hundred ninety-three pilots had an increased BMI > 25. This corresponds to a percentage of 72.8% in the overweight range (Table 1). Concerning demographic data − apart from the somewhat higher proportions of older employees and of working partners amongst pilots from the 1-week ROS − no (significant) differences were found between the two ROS.

**Table 1:** Demographic and lifestyle characteristics

	1-week ROS (*n* = 49)	4-month ROS (*n* = 352)	p
**Age,** years (SD)	50.3 (6.9)	48.2 (8.6)	0.052^1^
**BMI**, kg/m^2^(SD)	26.8 (3.1)	27.4 (3.7)	0.230^1^
≥ 25, n (%)	37 (75.5%)	256 (72.7%)	0.681^2^
**Partnership,** n (%)	48 (98.0%)	322 (91.5%)	0.111^2^
Working partner, n (%)	34 (70.8%)	183 (56.8%)	0.066^2^
Working hours/week (SD)	27.4 (11.0)	27.9 (13.0)	0.811^1^
**Children younger than 6 years,** n (%)	28 (57.1%)	180 (51.1%)	0.430^2^
**Lifestyle parameter**			
**Smoking,** n (%)			
*Current smoker*	7 (14.3%)	66 (18.8%)	0.538^2^
*Ex-smoker*	20 (40.8%)	155 (44.0%)	
*Never smoker*	22 (44.9%)	131 (37.2%)	
***Regular sport exercises,*** *n (%)*	14 (28.6%)	113 (32.1%)	0.619^2^
*During working period, mean h/week (SD)*	4.7 (3.6)	4.2 (2.0)	0.632^1^
*During holidays, mean h/week (SD)*	7.1 (5.3)	6.0 (3.6)	0.459^1^

^1^Student’s T test

^2^Chi-square test

In respect of lifestyle parameters, 18.2% indicated that they were current smokers and 38.2% have never smoked. 31.7% stated that they exercised regularly with distinctly less sport activities during their stays on board (Table 1). The lifestyle behavior was irrespective of the pilots’ ROS.

### Psychophysical stress

According to the answers in the online questionnaire, the main stressors in the pilot’s job were insufficiently predicable free time, irregular working hours and night work (Table 2). Pilots of the 4-month ROS experienced insufficiently predictable free time and long operation times at a stretch significantly more often as main stressors. On the other hand, pilots from the 1-week ROS rated working under wet and cold conditions as well as a high responsibility for safe navigation more often as relevant job-related stressors.

**Table 2:** Stressors in the pilot profession and accidents in respect of the rotation systems

	Total sample (*n* = 401)	1-week ROS (*n* = 49)	4-month ROS (*n* = 352)	p^1^
**Main stressors in the pilot profession,** n (%)				
*Not sufficiently predictable free time*	213 (53.1%)	5 (10.2%)	208 (59.1%)	**< 0.001**
*Irregular working hours*	208 (51.9%)	25 (51.0%)	183 (52.0%)	0.899
*Night work*	207 (51.6%)	29 (59.2%)	17 (50.6%)	0.258
*Insufficient sleeping times*	147 (36.7%)	21 (42.9%)	126 (35.8%)	0.336
*Non-professional bridge team*	85 (21.2%)	12 (24.5%)	73 (20.7%)	0.547
*Long operating times at a stretch*	66 (16.5%)	3 (6.1%)	63 (17.9%)	**0.037**
*Working under wet and cold conditions*	29 (7.2%)	11 (22.4%)	18 (5.1%)	**< 0.001**
*High responsibility for safe navigation*	20 (5.0%)	6 (12.2%)	14 (4.0%)	**0.013**
**Stand-by time** (8 days or 4 months respectively between two long vacations)				**< 0.001**
*Appropriate*	190 (47.4%)	41 (83.7%)	149 (42.3%)	
*Too long*	185 (46.1%)	6 (12.2%)	179 (50.9%)	
*Too short*	26 (6.5%)	2 (4.1%)	24 (6.8%)	
**Occupational accident with severe personal injury,** n (%)	79 (19.7%)	5 (10.2%)	74 (21.0%)	0.074
**Vessel damage,** n (%)	208 (51.9%)	37 (75.5%)	171 (48.5%)	**< 0.001**

^1^Chi-square test

Concerning the duration of assignments at a stretch, 50.9% of the pilots from the 4-month ROS regarded them as too long compared to 12.2% in the 1-week ROS (Fisher’s exact test; *p* < 0.001). The first-mentioned pilots judged durations of assignment over 5.6 weeks (SD 3.4) as optimal. 46.1% of the pilots (47.7% from the 4-month and 34.7% from the 1-week ROS; Fisher’s exact test; *p* = 0.037) complained that they were less able to meet their rest periods before night time due to private / family responsibilities. As a consequence, 83.3% stated that they had regularly neglected their private obligations (e. g. to take care of their family, particularly of their children) due to job assignments (84.5% from the 4-month and 71.9% from the 1-week ROS; Fisher’s exact test; *p* = 0.029). The majority complained of difficulties in reconciling work and private obligations (61.6%; 61.9% from the 4-month and 49.8% from the 1-week ROS; Fisher’s exact test; *p* = 0.034). Furthermore, in this study no pilot used the opportunity to add an additional stress or strain factor to the predetermined list as free text.

Among the pilots asked, 19.7% stated that throughout their working life they had experienced at least one occupational accident at work with personal damage and 51.9% damage to a vessel (ground contact, collision, etc.) in the context of their pilot activity (40% even more than once). While more 4-month ROS pilots reported to have had occupational accidents with severe personal injury, significantly more 1-week ROS pilots have had vessel damage in their job/career (Table 2).

### Psychophysical strain: fatigue and health of pilots

In the study sample, 68.1% of the pilots complained of sleeping disorders. According to the Epworth Sleepiness Scale, 41.9% of the pilots were regarded as having an elevated daily sleepiness without differences between the two ROS (Table 3).

**Table 3:** Sleeping problems and health status stated by the pilots from the two different ROS

	Total sample (*n* = 401)	1-week ROS (*n* = 49)	4-month ROS (*n* = 352)	p
**Sleeping disorders,** n (%)				
No	128 (31.9%)	18 (36.7%)	110 (31.3%)	0.440^2^
Yes	273 (68.1%)	31 (63.3%)	242 (68.7%)	
*Difficulty in falling asleep*	42 (10.5%)	3 (6.1%)	39 (11.1%)	
*Wake up at least once a night*	172 (42.9%)	19 (38.8%)	153 (43.5%)	
*Lie awake several times a night*	59 (14.7%)	9 (19.4%)	50 (14.2%)	
**Daytime sleepiness according to Epworth Sleepiness Scale**				
Point value, (SD)	8.8 (4.0)	8.1 (3.8)	8.9 (4.0)	0.207^1^
Point value > 11, n (%)	168 (41.9%)	16 (32.7%)	152 (43.2%)	0.162^2^
**Most common current diseases or injuries** (physician’s diagnosis), n (%)^3^				
Musculoskeletal disease in back, limbs or other part of the body	137 (34.2%)	20 (40.8%)	117 (33.2%)	0.205^2^
Cardiovascular disease	101 (25.2%)	7 (14.3%)	94 (26.7%)	0.098^2^
Injury due to an accident	73 (18.2%)	9 (18.4%)	64 (18.2%)	0.280^2^
Endocrine or metabolic disease	43 (10.7%)	4 (8.2%)	39 (11.1%)	0.419^2^
Neurological or sensory disease	42 (10.5%)	0	42 (11.9%)	**0.038**^2^
Mental disorder	22 (5.5%)	1 (2.0%)	21 (6.0%)	0.079^2^
**Work impairment due to these diseases** (subjective assessment), n (%)^3^				0.712^2^
No hindrance/ no diseases	267 (66.6%)	29 (59.2%)	238 (67.6%)	
Ability to do the job, but it causes some symptoms	109 (27.2%)	17 (34.7%)	92 (26.0%)	
***Sometimes*** slow down the work pace or change the work methods	18 (4.5%)	3 (6.1%)	15 (4.3%)	
***Often*** slow down the work pace or change the work methods	3 (0.7%)	0	3 (0.9%)	
Feel able to do only part-time work	1 (0.3%)	0	1 (0.3%)	
Entirely unable to work	3 (0.7%)	0	3 (0.9%)	

^1^Student’s T test

^2^Chi-square test

^3^according to WAI

### WAI questionnaire

According to the WAI questionnaire, most of the current diseases among pilots diagnosed by a physician were related to the musculoskeletal system at just below 35%, followed by cardiovascular diseases at 25%, and accidental injuries at just under 20%. These diseases were similarly frequently diagnosed in the two ROS. Two thirds of the study sample did not recognize any hindrances in their work due to these diseases, and 27% felt able to do their job, although it causes some symptoms (Tab. 3).

79.8% of the pilots indicated that they had psychological demands at work (83.2% vs. 55.1% among pilots of the 4-month and 1-week ROS respectively). The majority of the pilots surveyed estimated their current ability to work to be better than average in comparison to the best score ever achieved. Irrespective of the ROS, 74.5% reported a rather good to very good ability to work in terms of physical and 85.5% in terms of mental work demands. 4.3 and 2.0% of the pilots assessed a rather to very poor current work ability with regard to the respective physical and mental demands of the work (Table 4).

**Table 4:** Demands of the work and work ability (according to WAI - questionnaire) in respect to the ROS

	Total sample (*n* = 401)	1-week ROS (*n* = 49)	4-month ROS (*n* = 352)	p
**Demands of the work**, n (%)				
Psychological	320 (79.8%)	27 (55.1%)	293 (83.2%)	**< 0.001**^2^
Physical	0	0	0	
Physical and psychological	81 (20.2%)	22 (44.9%)	59 (16.8%)	
**Current work ability compared to highest work ability ever scored,** score (SD) (from 0 (completely unable to work) to 10 (work ability at its best))	7.8 (1.6)	8.0 (1.1)	7.8 (1.7)	0.337^1^
**Current work ability with respect to**				
***Physical demands of the work***				0.688^2^
Rather good / very good	299 (74.5%)	38 (77.6%)	261 (74.2%)	
Moderate	85 (21.2%)	9 (18.3%)	76 (21.5%)	
Rather poor / very poor	17 (4.3%)	2 (4.1%)	15 (4.3%)	
***Mental demands of the work***				0.866^2^
Rather good / very good	343 (85.5%)	43 (87.8%)	300 (85.2%)	
Moderate	50 (12.5%)	6 (12.2%)	44 (12.5%)	
Rather poor / very poor	8 (2.0%)	0	8 (1.7%)	

^1^Student’s T test

^2^Chi-square test

Irrespective of the ROS, 35.9% had no sick leave during the past year. The majority reported sick leave from 0 to 9 days (74.3%) and 1.2% were ill for 100–365 days. 93.3% predicted a relatively certain work ability in 2 years with no differences between the ROS. Concerning the mental capacities, more than 70% of both ROS stated that they were often/rather often able to enjoy their daily tasks or they had been active and alert during the last 3 months. However, significantly fewer pilots from the 4-month ROS indicated that they were hopeful about their future (Table 5).

**Table 5:** Sick leave, mental capacities and ability to work (according to WAI - questionnaire) in respect to the ROS

	Total sample (*n* = 401)	1-week ROS (*n* = 49)	4-month ROS (*n* = 352)	p
**Sick leave last year** (12 months)				0.593^2^
None at all	144 (35.9%)	17 (34.7%)	127 (36.1%)	
1–9 days	158 (39.4%)	21 (42.9%)	137 (38.9%)	
10–99 days	94 (23.4%)	11 (22.4%)	83 (23.6%)	
> 100 days	5 (1.3%)	0	5 (1.4%)	
**Estimation of the own work ability in 2 years**				0.452^2^
Relatively certain	374 (93.3%)	46 (93.9%)	328 (93.2%)	
Not certain	24 (6.0%)	2 (4.1%)	22 (6.3%)	
Unlikely	3 (0.7%)	1 (2.0%)	2 (0.5%)	
**Mental capacities**				
**Ability to enjoy the regular daily activities**				0.142^2^
*Often / rather often*	307 (76.5%)	43 (87.8%)	264 (75.0%)	
*Sometimes*	68 (17.0%)	5 (10.2%)	63 (17.9%)	
*Rather seldom / never*	26 (6.5%)	1 (2.0%)	25 (7.1%)	
**Considering the last 3 months:** **Have you been active and alert?**				0.513^2^
*Often / rather often*	289 (72.0%)	39 (79.6%)	250 (71.0%)	
*Sometimes*	76 (19.0%)	8 (16.3%)	68 (19.3%)	
*Rather seldom / never*	36 (9.0%)	2 (4.1%)	34 (9.7%)	
**Considering the last 3 months:** **Have you felt yourself to be full of hope about the future?**				
				**0.007**^2^
*Continuously/ rather often*	288 (71.5%)	38 (77.6%)	250 (71.0%)	
*Sometimes*	81 (20.2%)	8 (16.3%)	73 (20.7%)	
*Rather seldom / never*	32 (8.3%)	3 (6.1%)	29 (8.3%)	
**Ability to work according to WAI**				0.432^2^
Very good	122 (30.4%)	18 (36.7%)	104 (29.5%)	
Good	188 (46.9%)	22 (44.9%)	166 (47.2%)	
Moderate	77 (19.2%)	9 (18.4%)	68 (19.3%)	
Critical	14 (3.5%)	0	14 (4.0%)	
***Point value***, mean (SD)	40.2 (5.7)	40.9 (4.8)	40.2 (5.8)	0.362^1^

^1^Student’s T test

^2^Chi-square test

The overall evaluation of the WAI questionnaire showed good to very good work ability at 77.3%. 19.2% had a moderate and 3.5% a critical work ability. By trend, the 1-week ROS pilots were more often allocated to the group with very good work ability. On the basis of the WAI median, pilots from the 4-month ROS had a not significantly higher risk for elevated work inability (OR 1.07; 95% CI 0.59–2.00). After adjustment for age, working years as a pilot, examination time (during working phase vs. vacation phase) and ESS, only increased daily sleepiness was associated with a higher work inability (*p* < 0.001).

## Discussion

This study aimed to assess stress and strain as well as the work ability of maritime pilots by using standardized questionnaires, taking into account the different rotation systems. In 2015 Main and Chambers published a review about fatigue and coping strategies in maritime pilotage. In the time frame from 1977 to 2014, they identified only 18 studies and concluded that most of these available studies were rather old and often relied on very small study populations [5]. The present study is the first one dealing with stress and strain as well as the work ability of maritime pilots by using standardized questionnaires.

Within the past decades, pilotage has undergone extensive development: firstly, the working conditions of the pilots have changed, e. g. due to the growing size of container ships, higher technical requirements, or newly formed training paths due to stagnating numbers of newcomers [23]. Secondly, the awareness of work-life balance has changed, which may lead to a decrease in job satisfaction [24]. These developments should be taken into consideration when assessing the current job-related psychophysical stress and strain of pilots. This also highlights the finding that the old available studies are not suitable for judging the current circumstances in the pilots’ profession and there is a need to gain new knowledge.

In the present study, most pilots evaluated irregular working hours including night work as the main stressor of their job resulting in unplannable family time. On the one hand, a majority of pilots (especially from the 4-month ROS) experienced relevant psychological demands in their workplace and stated that they were less often full of hope about the future. This may indicate their subjective perception of particularly high work-related mental stress. On the other hand, more than 80% of the pilots stated having regularly neglected their private obligations. This suggests that many pilots had difficulties in reconciling work and private requirements, which can be seen as a sign of disturbed work-life balance. The well-known finding that one of the major stress factors for seafarers on board is the long-term separation from their family and the loneliness on the high seas should emphasize the importance of the family for pilots [12, 25]. Maritime captains normally have contracts for at least 3–4 months at a stretch; their urgent need for more time for the family after a life at sea as a captain is an important reason for an occupational change in favor of a career as a pilot [26, 27].

Even though a lot of pilots mentioned their subjective stress due to difficulties in reconciling working life and family life, the stress level varied amongst the ROS. Pilots from the 4-month ROS experienced insufficiently predictable free time and long operation times at a stretch significantly more often as main stressors. This is in line with the observation within the OECD study “Employment Outlook” [28], which concludes that unpredictable working hours correlate with lower family satisfaction. Besides their higher mental load through long-term assignments, this could possibly lead to a higher dissatisfaction with their work situation among pilots with 4-month ROS compared to their colleagues. Pilots from the 1-week ROS stated working under wet and cold conditions more often as the main stressor as they need to work outside during berthing maneuvers. Moreover, the fact that the pilots of the 1-week ROS tended to more often have children younger than 6 years and a working partner may indicate better reconciliation of work and family in this pilot group.

High stress load in the workplace can lead to psychophysical exhaustion and fatigue [29]. The present study also examined the occurrence of daily sleepiness. Higher results for daily sleepiness were found among pilots in comparison to professional groups with a higher risk of fatigue (e. g. truck and bus drivers or shift worker) [30–32]. Although significantly more pilots in 4-months ROS experienced psychological demands of the work, no difference was observed in the daily sleepiness between the employees of the two different ROS. Ferguson et al. [10] already described the positive effects of short, irregular sleep opportunities at sea on the alertness of marine pilots. For that reason, it is recommended to counteract fatigue through short naps in cases where pilots are waiting at the pilot station for the next assignment.

Generally, psychophysical exhaustion can increase the risk of maritime disasters or accidents [33, 34]. In this study, 4-month ROS pilots distinctly more often reported having had occupational accidents with severe personal injuries that may be explained by their pilotage district in the environmentally rougher coastal area. Furthermore, both boarding and leaving the vessel require climbing on the pilot ladder at the outer wall of the ship, which is physically stressful and potentially hazardous. In contrast, significantly more 1-week ROS pilots stated having had vessel damage. This was expected since grounding is a major risk within the harbor basins.

Specific causes of occupational accidents and vessel damage have not been further investigated in this study. According to a Belgian study, the accidents during pilotage were mostly due to the harsh environment (wind speed, state of the sea, poor visibility); Human factors accounted for 11.7% (stress, sleep deprivation, bad physical condition), with only 2.9% of the accidents caused by not enough sleep [11]. In light of the high percentage of daily sleepiness among the pilots examined, further research is of high importance to investigate the extent to which human factors are causal to occupational accidents and maritime disasters during pilotage.

Moreover, due to the unpredictability of the assignments and the night work, an irregular and possibly not healthy diet is typical for pilots. This hypothesis adds support to the present study as more than 70% of the study sample was classified as overweight. This is in line with the results of the review from Main and Chambers [5] on the factors affecting maritime pilots’ health and well-being. The “healthy worker effect” needs to be considered to ensure that there is no selection bias. As pilots are required to have regular medical check-ups concerning their health ability to work at sea, the study population should have a better general state of health in comparison to the general population [35].

In contrast, Main and Chambers [5] observed that pilots are at a significantly higher risk of health impairment than the general population; particularly cardiovascular diseases, mental illnesses (sleep disorders, depression, burnout), and accidents were frequently found in the 18 studies observed. Among pilots examined in this study, the most current diseases were related to the musculoskeletal system at just below 35%, followed by cardiovascular diseases at 25%, and accidental injuries at just under 20% − in similar frequency between the two ROS.

An unfavorable lifestyle and obesity have also been repeatedly discussed as a major risk factor for cardiovascular diseases in seafaring populations. Several studies have consistently shown that obesity, smoking, high lipid levels in the blood, lack of exercise, and unhealthy eating habits are much more common in seafarers than in the general population [3, 36, 37]. In the present study, particularly overweight was more prominent among pilots compared to that of the male German general population (73.1%. vs. 62.1% [38]). Furthermore, no differences in lifestyle factors were found between the two ROS. Generally, it is assumed that job-related stress is associated with an unhealthy lifestyle [39]. Assuming higher psychological demands of the work among pilots of the 4-month ROS, such association, however, cannot be shown in this study.

To assess the stress and strain of maritime occupations, a pilot-specific questionnaire was developed based on an established seafaring-specific questionnaire [12]. This was about seafarers’ stress and strain and has previously been used several times in various maritime studies [13, 14]. The questionnaire of this study was created by the heads of some pilots’ associations and several pilots. Finally, it was tested and improved in a pilot study. In the online portal, all pilots had the opportunity to comment and to add some further aspects in free text. Overall, a sufficiently high representativeness was assumed for the assessment of the pilots’ stress and strain, since several pilots answered the above-mentioned maritime specific questionnaire and they had the opportunity to add important aspects within the supplementary free text.

According to the WAI in this study, the work ability of pilots showed no differences in comparison to those of other land-based study populations (teachers, office workers, executives) [40]. In addition, no significant differences in the work ability were observed between the pilots of the two different ROS.

In total, this study revealed, on the one hand, more often job-related mental demands, disturbed work-life balance and longer operation times at a stretch among 4-month ROS, indicating significantly higher job stress. On the other hand, this higher stress level does not lead to more sleepiness or reduced work ability in this occupational group. This suggests that the pilots of the 4-month ROS might be highly adapted to their working situation aboard. In view of the pilots’ higher average age of 48.5 years, it can be assumed that their job activity was carried out on average for at least 15 years. Moreover, since a switch between the two ROS almost never occurs, the pilots are not familiar with the alternative rotation system and have likely adjusted to their working conditions, so that, despite an increased work-related stress level in the 4-month ROS, there is no increased risk of daytime sleepiness or inability to work.

Another explanation for the missing correlation could be that the pilots with the 4-month ROS (who were obviously much more often stressed) more frequently downplayed both their sleepiness and their inability to work in the sense of social desirability. Furthermore, according to the assessment of many pilots, the working conditions for a 4-month deployment are regarded as given and unchangeable. Thus, it can be assumed that these pilots as freelancers perceive safety-related restrictions due to increased sleepiness or inability to work as a threat to their existence. Accordingly, the examiners repeatedly observed emotional discussions about the need to change the ROS, especially among pilots with the 4-month ROS. In addition, it cannot be excluded that many pilots with severe sleepiness or inability to work − phenomena that are mainly suspected in the 4-month ROS − did not take part in this study. As a result, these pilots could be underrepresented in this survey in the sense of a selection bias.

### Strengths and weaknesses of the study

As a limitation, the present study focused on sea, canal and port pilots in Germany. It is yet unclear how far the results are transferable to other international pilot systems. Furthermore, the freelancer status of pilots has to be taken into consideration as it makes it scarcely possible to compare them with other land-based occupational groups. Additionally, only a small group of 1-week ROS pilots were present in this study. However, since only the port pilots’ associations practice the latter ROS and there are far less port pilots than river or sea pilots, a considerably smaller number of 1-week ROS was expected. The proportion of participants was similar in each ROS. The demographic data of the participating pilots were also not different from those of the total pilot group, indicating that no bias or underrepresentation was present.

The participation rate of 46.9% is usual in online surveys, and a selection bias cannot be ruled out. There are no current studies on the pilots’ working conditions, so there is no experience with the willingness of this professional group to participate in studies. Finally, the questionnaire used on stress and strain was not standardized as no suitable instrument is available to assess the pilot-specific work situation. Thus, a pilot specific questionnaire was developed based on a repeatedly used questionnaire about the seafarers’ stress and strain and on the statements by several pilots. As in this study no pilot used the opportunity to add an additional stress or strain factor as free text to the predetermined list, this questionnaire is regarded as complete and representative for assessing the pilots’ stress and strain – especially in light of the satisfying internal consistency with a Cronbach’s alpha of 0.76.

## Conclusions

Altogether, this study suggests that pilots from the 4-month ROS have higher job stress than 1-week ROS pilots, which is likely to be due to their long-term assignments and the subsequent lack of predictability in their everyday life. Therefore, intervention measures with shortened ROS (preferably no longer than 6 weeks) amongst sea and canal pilots’ associations should be tested in respect of benefit, practicability and acceptance by the pilots.

## Data Availability

The datasets used and/or analyzed during the current study are available from the corresponding author on reasonable request.

## Abbreviations

BMI: Body mass index
CI: Confidence interval
ESS: Epworth Sleepiness Scale
OR: Odds ratio
ROS: Rotation systems
SD: Standard deviation
WAI: Work Ability Index
